# Post-discharge mortality in adult patients hospitalized for tuberculosis: a prospective cohort study

**DOI:** 10.1590/1414-431X2023e12236

**Published:** 2023-01-27

**Authors:** A.M. Müller, C.S. Osório, R.V. Figueiredo, D.R. Silva, P.T.R. Dalcin

**Affiliations:** 1 Programa de Pós-Graduação em Ciências Pneumológicas, Universidade Federal do Rio Grande do Sul, Porto Alegre, RS, Brasil; 2 Faculdade de Medicina, Universidade Federal do Rio Grande do Sul, Porto Alegre, RS, Brasil; 3 Programa de Pós-Graduação em Ciências Pneumológicas, Serviço de Pneumologia, Hospital de Clínicas de Porto Alegre, Faculdade de Medicina, Universidade Federal do Rio Grande do Sul, Porto Alegre, RS, Brasil

**Keywords:** Tuberculosis, Mortality, Risk factors, Post-discharge, Hospitalization

## Abstract

Determining outcomes and predictors of mortality following discharge from tuberculosis (TB) hospitalization is crucial to establish health policies. The objective of this study was to analyze outcomes and, secondarily, predictors of mortality following discharge from TB hospitalization. This was a prospective cohort study of patients diagnosed with TB (all forms) discharged from the hospital who began treatment during hospitalization. Out of 169 subjects included, 38 died during the 13-months of follow-up, within a median of 3 months (95%CI: 1.49-4.51). In the multivariate analysis, the variables independently associated with death were age (HR=1.04, 95%CI: 1.01-1.06, P=0.001), presence of sputum production (HR=2.18, 95%CI: 1.09-4.34, P=0.027), and Charlson Comorbidity Index (HR=1.19, 95%CI: 1.04-1.36, P=0.015). In conclusion, post-discharge mortality in subjects hospitalized for TB was 22.5%, with mean survival of 4.6 months. The mortality was higher in older subjects, in those who reported sputum production, and in those with a high comorbidity index.

## Introduction

Tuberculosis (TB) is one of the top 10 causes of death worldwide. In 2018, 10.4 million people fell ill with TB, and 1.2 million died from the disease. Over 95% of TB deaths occur in low- and middle-income countries (1). TB mortality shows a tendency for reduction in Brazil. In 2004, the country had a mortality coefficient of 2.8 cases per 100,000 inhabitants (4981 deaths) (2) and, in 2016, it was reduced to 2.2 per 100,000 inhabitants (4.483 deaths), representing a reduction of 15.4% (3). In the state of Rio Grande do Sul, Southern Brazil, the mortality rate in 2017 was 2.38 per 100,000 inhabitants, and in its capital city, Porto Alegre, the mortality rate was 4.4 per 100,000 inhabitants (4).

Although TB is a curable disease, it can progress to more severe forms, leading to poor prognosis and high mortality rates. It is known that the in-hospital mortality of patients with TB remains high. However, mortality remains high even after hospital discharge, with a higher rate in the first months of treatment (5-7).

Main risk factors for TB mortality are diagnostic delay, presence of comorbid conditions, smoking, drug use, previous TB, advanced age, male gender, and HIV-coinfection (6-10). Also, in the first year after TB diagnosis, other conditions independently associated with increased mortality were treatment by a private provider, not using directly observed therapy (DOT), poor treatment adherence, and belonging to indigenous ethnicity (6,11).

A systematic review showed that the risk factors for death were HIV-coinfection, advancing immunosuppression, smear-negative disease, and malnutrition. In regions of low TB incidence and HIV prevalence, risk factors include non-infective comorbidities, sputum smear-positive disease, and alcohol and substance misuse (12).

A significant proportion of TB patients must be hospitalized. Some of these hospitalizations relate to cases that are more severe (13,14). A retrospective cohort study of patients hospitalized for TB in Southern Brazil identified a one-year mortality of 31.8% in post-discharge subjects (15). Determining outcomes and the factors associated with them following discharge from TB hospitalization is crucial to better understand the potential impact of the disease, which may help establish health policies to improve survival.

The objective of this study was to assess post-discharge mortality (time to death) in the 13 months of follow-up of a cohort of adult subjects hospitalized for TB and who began treatment for TB during hospitalization. Secondarily, the study aimed to evaluate clinical factors associated with death.

## Material and Methods

This was a prospective cohort study after hospital discharge of patients who were diagnosed with TB and began treatment during hospitalization. It was conducted at Hospital de Clínicas de Porto Alegre (HCPA), located in Porto Alegre, southern Brazil. The HCPA is a general, tertiary care, university-affiliated hospital. This work was part of a larger research project for educational strategy intervention on the post-discharge management of tuberculosis diagnosed in the hospital.

The study was approved by the HCPA Ethics Committee (protocol number 13-0192) and by Plataforma Brasil (protocol number 10955112.0.0000.5327). All methods were performed in accordance with the international and national standards for clinical study in humans. The study was performed in accordance with the Declaration of Helsinki. Written informed consent was obtained from all participants at recruitment and patients were followed up for 13 months after hospital discharge.

The study population consisted of patients aged 18 years or older with newly diagnosed TB (all forms) who were hospitalized for the disease. We included only patients who began treatment for TB after hospitalization. A new case of TB was defined as patients who had never been treated for TB or had taken anti-TB drugs for less than 1 month (16). Hospitalization was defined as a hospital stay ≥24 h in any health care unit of HCPA. Pulmonary TB was diagnosed according to any of the following criteria established in the Brazilian Guidelines for TB (17): 1) detection by a direct test (Ziehl-Neelsen (ZN) method) - two positive samples; 2) detection by a direct test (ZN method) - one positive sample and a culture result positive for *Mycobacterium tuberculosis* (in Löwenstein-Jensen (LJ) medium); 3) detection by a direct test (ZN method) - one positive sample and radiological findings compatible with TB; 4) only a positive culture result for *Mycobacterium tuberculosis* (in LJ medium); 5) presence of clinical, epidemiologic and radiographic findings compatible with TB. The diagnosis of extra-pulmonary TB was based on clinical and/or complementary tests according to the location of TB. GeneXpert MTB/RIF was not used for diagnosis, because it was not available during the study. The exclusion criteria were as follows: cases in which the diagnosis was subsequently changed; cases who had started treatment before hospitalization; and patients who died immediately after inclusion in the study before being discharged from the hospital.

Patients were identified based on the prescription of anti-TB drugs and from the issuance of the National System of Information on Notifiable Diseases (SINAN) form. SINAN is a database from the Brazilian government that stores information concerning all notifiable infectious and contagious diseases. In HCPA, the electronic prescription of anti-TB drugs automatically generates the SINAN form, so all patients who begin the treatment can be identified.

Patients were invited to participate in the study, and after they signed the informed consent form, they were included in the study. Patients were interviewed by the investigators, who completed a standardized questionnaire including the following items: demographic data (age, sex, race, marital status, years of schooling, and standard minimum wage), previous TB, symptoms at admission, smoking status, alcoholism, and drug use. Other information was found in patient records: length of hospitalization, ICU admission, clinical form of TB, HIV infection, presence of comorbidities, diagnostic methods, drug regimen, and outcome after discharge.

The primary outcome of the study was post-discharge mortality (time to death) in the 13 months of follow-up. Death was defined as a patient who died for any reason during TB treatment, the standard WHO definition (18). Outcomes were obtained by reviewing patient charts, by searching the SINAN database, or by calling the primary health care clinics where the patients were being followed. The probable cause of death of each individual was obtained from the Mortality Information System and, for those that died in readmission to HCPA, from the patient electronic chart.

Data analysis was performed using the IBM SPSS Statistics version 22.0 (USA). We carried out a descriptive analysis of the study variables in each group (survival *vs* death). Qualitative data are reported as number of cases (%) and proportion. Quantitative data are reported as means±SD, or median with interquartile range (IQR). The distribution of the data was evaluated through the Shapiro-Wilk test. Proportions were compared with the chi-squared test for categorical variables and the Student's *t*-test for continuous variables. Kaplan-Meier curves, compared to log-rank tests, were used for cumulative survival analyzes. P-values <0.05 were considered significant. Survival analysis was performed using Cox proportional risk regression models: i) events were defined as time post-discharge to death; ii) censored data were used when the event did not occur at the end of the follow-up period. All parameters with a P-value <0.10 in the univariate analysis were included in a multivariate model and considered statistically significant if the overall P-value was <0.05. The analysis supported the hypothesis of proportional risk and the variables that did not meet this criterion were educational level and cure.

Based on data from a previous study (19), which informed a mortality rate after discharge from TB in Porto Alegre of 18.8%, we estimated a sample size of 189 patients, under the assumptions of a type I error (two-sided) of 1% and total confidence interval amplitude of 0.15.

## Results

**Figure 1:**
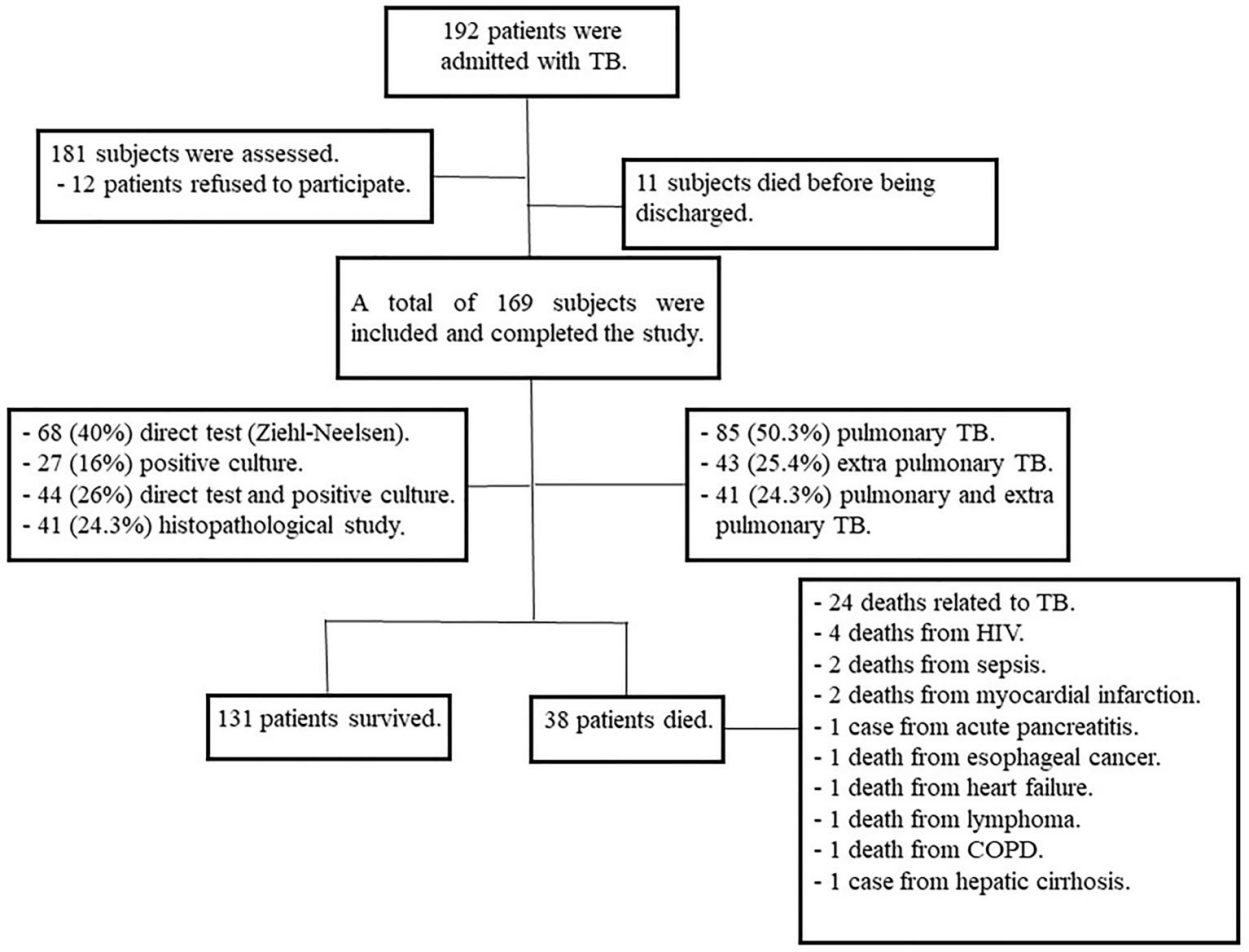
Flow diagram of the study.

In the 36 months of the study, 192 patients were admitted to the hospital with TB. Eleven subjects died before being discharged. We assessed 181 subjects, but 12 patients refused to participate. Of those, 169 were included and completed the study (Figure 1).

Demographic characteristics of all patients and comparison between groups (survival and death) are presented in Table 1. The mean age of all patients was 46.2±15.6 years and women outnumbered men (62.7 *vs* 37.3%). Most subjects were white (60.9%), 50.3% had less than 8 years of schooling, and 25.4% had a very low-income level (≤1 standard minimum wage). Before discharge, the median length of hospital stay was 19 days (interquartile range - IR: 11-29) and 23 subjects (13.6%) had intensive care unit admission. The most common symptoms were loss of weight (83.4%), fever (75.1%), and cough (66.3%). There was a high proportion of people that were currently smokers (34.9%), and a significant proportion of current alcoholism (15.4%) and current illicit drug use (15.4%). People in the study had a high number of comorbidities, with Charlson Comorbidity Index of 4.1±2.8. There were 87 (51.5%) people living with HIV in the study and 15 (8.9%) were diagnosed during hospitalization. Sputum smear was positive in 112 (66.3%) cases, culture was positive in 71 (42%), and chest X-ray interpretation suggested pulmonary TB in 142 (84%) subjects. Eighty-five (50.3%) of 169 subjects had only pulmonary TB, 42 (24.9%) had only extrapulmonary TB, and 42 (24.9%) had association of pulmonary and extrapulmonary TB. The treatment defaulters were 11 (6.5%) and the cure rate was 69.8% (118 subjects).

**Table 1:** Demographic and clinical characteristics of study participants.

Variables	Total (N=169)	Survival (n=131)	Death (n=38)	P-value
Demographics				
Male	106 (62.7)	82 (62.6)	24 (63.2)	1.000
Age, years	46.2±15.6	44.6±15.3	52.0±15.4	**0.009**
White	103 (60.9)	81 (61.8)	22 (57.9)	0.661
Educational level, <8 years of schooling	85 (50.3)	59 (45.0)	26 (68.4)	**0.011**
Marital status				**0.048**
Single	65 (38.5)	47 (35.9)	18 (47.4)	
Married	75 (44.4)	63 (48.1)	12 (31.6)	
Divorced/Separated	16 (9.5)	14 (10.7)	2 (5.3)	
Widowed*	13 (7.7)	7 (5.3)	6 (15.8)	
Income level per month, ≤1 standard MW*	43 (25.4)	30 (22.9)	13 (34.2)	0.159
Clinical				
Previous TB diagnosis	16 (9.5)	12 (9.2)	4 (10.5)	0.505
Length of hospital stay, days	19 [11-29]	18 [10-29]	24 [12-43]	0.083
ICU admission	23 (13.6)	18 (13.7)	5 (13.2)	1.000
Dyspnea	82 (48.5)	67 (51.1)	15 (39.5)	0.205
Cough	112 (66.3)	86 (65.6)	26 (68.4)	0.750
Sputum	71 (42.0)	49 (37.4)	22 (57.9)	**0.024**
Fever	127 (75.1)	101 (77.1)	26 (68.4)	0.276
Hemoptysis	27 (16.0)	22 (16.8)	5 (13.2)	0.802
Chest pain	70 (41.4)	55 (42.0)	15 (39.5)	0.782
Night sweats	98 (58.0)	75 (57.3)	23 (60.5)	0.719
Loss of weight	141 (83.4)	106 (80.9)	35 (92.1)	0.102
Current smoker	59 (34.9)	41 (31.3)	18 (47.4)	0.067
Current alcoholism	26 (15.4)	19 (14.5)	7 (18.4)	0.556
Current illicit drug use	26 (15.4)	18 (13.7)	8 (21.1)	0.271
Charlson Comorbidity Index	4.1±2.8	3.8±2.7	5.2±2.8	**0.003**
HIV status	87 (51.5)	63 (48.1)	24 (63.2)	0.102
Smear-positive sputum	112 (66.3)	87 (66.4)	25 (65.8)	0.943
Positive culture	71 (42.0)	50 (38.2)	21 (55.3)	0.060
Chest X-ray**	142 (84.0)	110 (84.0)	32 (84.2)	0.972
Pulmonary tuberculosis	85 (50.3)	65 (49.6)	20 (52.6)	0.744
Treatment with basic drug regimen***	161 (95.3)	127 (96.9)	34 (89.5)	0.077
DOTS or supervised treatment	101 (59.8)	82 (62.6)	19 (50.0)	0.163
Treatment default	11 (6.5)	11 (8.4)	-	-
Cure	118 (69.8)	118 (90.1)	-	-

Data are reported as n (%), means±SD, or median [IQR]. P-values in bold are statistically significant. Chi-squared test was used for categorical variables and the Student's *t*-test was used for continuous variables. MW: minimum wage; TB: tuberculosis; ICU: intensive care unit; HIV: human immunodeficiency virus; DOTS: directly observed treatment short course. *1 standard minimum wage corresponded to approximately US$241. **Interpretation suggesting pulmonary TB. ***Basic drug regimen (rifampicin, isoniazid, ethambutol, and pyrazinamide).

Thirty-eight TB patients died during the study period. The probable cause of death was related to TB in 24 cases, to HIV in 4 cases, to sepsis in 2 cases, to myocardial infarction in 2 cases, to acute pancreatitis in 1 case, to esophageal cancer in 1 case, to heart failure in 1 case, to lymphoma in 1 case, to chronic obstructive pulmonary disease in 1 case, and to hepatic cirrhosis in 1 case. Of those who died, the mean age was 52.0±15.4 years *vs* 44.6.0±15.3 years of survivors (P=0.009). Also, of the subjects who died, 24 (63.2%) were female *vs* 14 (36.8%) males (P=1.00). The proportion of subjects with educational level <8 years of schooling was higher in those who died (68.4%) than in those who survived (45%; P=0.011). Also, the proportion of subjects with sputum was higher in those who died (57.9%) than in those who survived (37.4%, P=0.024), and Charlson Comorbidity Index was higher in those who died (5.2±2.8) than in those who survived (3.8±2.7, P=0.003).

**Figure 2:**
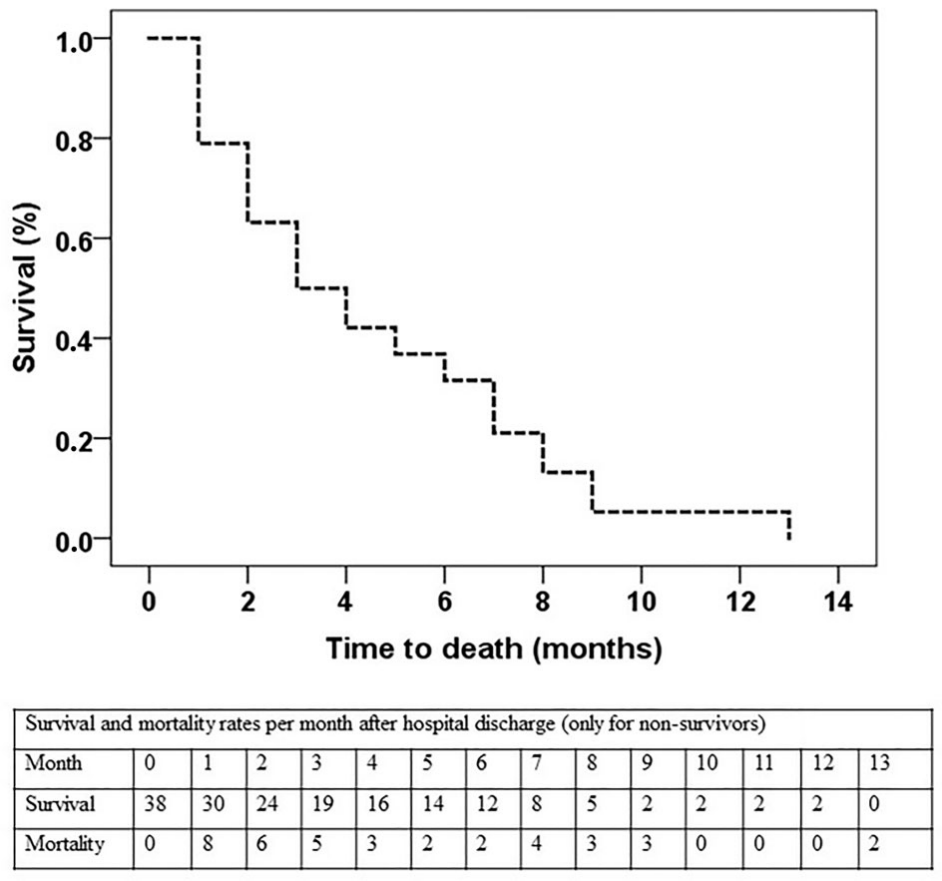
Kaplan-Meier survival curve for non-survivors. Out of 169 patients, 38 died within a mean of 4.6 months (95%CI: 3.49-5.62) and a median of 3 months (95%CI: 1.49-4.51). The table shows survival and mortality rates per month after hospital discharge (only non-survivors).

The Kaplan-Meier survival curve is shown in Figure 2. Of the 169 patients, 38 died within a mean of 4.55 months (95%CI: 3.49-5.62) and a median of 3 months (95%CI: 1.49-4.51).

Univariate and multivariate Cox regression analysis for post-discharge mortality is presented in Table 2. The following variables with P<0.10 in the univariate Cox regression model analysis were included in the multivariate analyses: age (HR=1.03, 95%CI: 1.01-1.05, P=0.008), length of hospital stay (HR=1.01, 95%CI: 0.99-1.02, P=0.067), presence of sputum (HR=2.02, 95%CI: 1.06-3.84, P=0.033), current smoker (HR=1.73, 95%CI: 0.91-3.26, P=0.093), Charlson Comorbidity Index (HR=1.18, 95%CI: 1.04-1.34, P=0.011), and positive culture (HR=1.84, 95%CI: 0.97-3.48, P=0.062).

**Table 2:** Cox regression analysis for post-discharge mortality in adult patients hospitalized for tuberculosis.

Variables	Univariate analysis****		Multivariate analysis	
	HR (95%CI)	P-value	HR (95%CI)	P-value
Demographics				
Male	1.05 (0.54-2.02)	0.892		
Age, years	1.03 (1.01-1.05)	0.001	1.04 (1.01-1.06)	**0.001**
White	0.89 (0.47-1.71)	0.746		
Educational level, <8 years of schooling	2.42 (1.22-4.79)	0.012	1.53 (0.75-3.13)	0.247
Income level per month, ≤1 standard MW*	1.66 (0.85-3.25)	0.137		
Clinical				
Previous TB diagnosis	1.19 (0.42-3.34)	0.747		
Length of hospital stay, days	1.01 (0.99-1.02)	0.067	1.01 (1.00-1.02)	0.182
ICU admission	0.93 (0.36-2.39)	0.882		
Dyspnea	0.66 (0.35-1.27)	0.217		
Cough	1.08 (0.55-2.14)	0.825		
Sputum	2.02 (1.06-3.84)	0.033	2.18 (1.09-4.34)	**0.027**
Fever	0.70 (0.35-1.38)	0.301		
Hemoptysis	0.74 (0.29-1.89)	0.529		
Chest pain	0.91 (0.47-1.74)	0.769		
Night sweats	1.16 (0.60-2.22)	0.659		
Loss of weight	2.44 (0.75-7.93)	0.139		
Current smoker	1.73 (0.91-3.26)	0.093	1.45 (0.73-2.91)	0.291
Current alcoholism	1.23 (0.54-2.78)	0.628		
Current illicit drug use	1.52 (0.70-3.32)	0.291		
Charlson Comorbidity Index	1.18 (1.04-1.34)	0.011	1.19 (1.04-1.36)	**0.015**
HIV status	1.66 (0.86-3.21)	0.131		
Smear-positive sputum	0.94 (0.48-1.84)	0.858		
Positive culture	1.84 (0.97-3.48)	0.062	1.95 (0.99-3.85)	0.054
Chest X-ray**	0.96 (0.40-2.30)	0.932		
Pulmonary tuberculosis	1.11 (0.59-2.1)	0.757		
Treatment with basic drug regimen***	0.43 (0.15-1.2)	0.107		
DOTS or supervised treatment	1.53 (0.81-2.89)	0.191		

Data are reported as HR (95%CI). Bold type indicates significance. HR: hazard ratio; CI: confidence interval; MW: minimum wage; TB; tuberculosis; ICU: intensive care unit; HIV: human immunodeficiency virus; DOTS: directly observed treatment short course. *1 standard minimum wage corresponds to approximately US$241. **Interpretation suggesting pulmonary TB. ***Basic drug regimen (rifampicin, isoniazid, ethambutol, and pyrazinamide). ****All parameters with a P-value <0.10 in the univariate analysis were included in the multivariate model and considered statistically significant if the overall P-value was <0.05.

**Figure 3:**
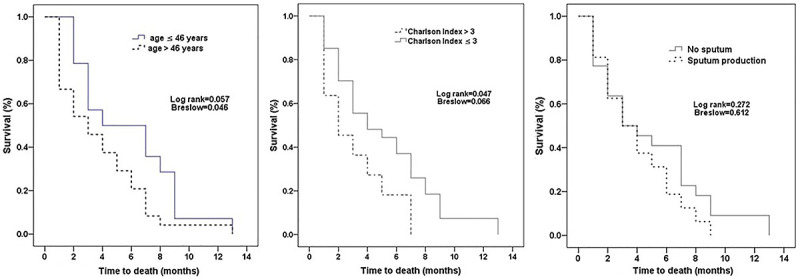
Kaplan-Meier survival curve for the variables independently associated with death in the multivariate Cox regression model analysis (from left to right): age, Charlson Comorbidity Index, and presence of sputum production.

In the multivariate Cox regression model analysis (Figure 3), the variables independently associated with death were age (HR=1.04, 95%CI: 1.01-1.06, P=0.001), sputum production (HR=2.18, 95%CI: 1.09-4.34, P=0.027), and Charlson Comorbidity Index (HR=1.19, 95%CI: 1.04-1.36, P=0.015).

## Discussion

This prospective study evaluated post-discharge mortality in the 13 months of follow-up of a cohort of adult subjects hospitalized for TB and who began treatment for the disease during hospitalization. Out of 169 subjects included in the study, 38 (22.5%) died within a median time of 3 months. The variables independently associated with death were age, presence of sputum production, and Charlson Comorbidity Index. The mortality was higher in older subjects, in those who reported sputum production, and in those with high comorbidity index.

It is worth noting that the population of the study was of subjects in a vulnerable social situation. Most subjects (50.3%) had less than 8 years of schooling and 25.4% had a very-low income level (≤1 standard minimum wage). There was a high proportion of people that were current smokers (34.9%), and a significant proportion of current alcoholism (15.4%) and current illicit drug use (15.4%). Also, people in the study had a high number of comorbidities, with Charlson score of 4.14 points, and there were 87 (51.5%) people living with HIV. It is well-known that TB is associated with overcrowding, poor sanitation and housing conditions, and poor access to health services (20-23). TB mortality rates are also affected by socioeconomic inequalities. People of lower socioeconomic status are likely to have worse treatment outcomes than the general population (24-26).

In the current study, most deaths (63%) were related to TB. There is a paucity of studies that describe the cause of post-discharge mortality in subjects hospitalized for TB. In a previous retrospective study, Silva et al. (19) demonstrated a mortality rate (18.8%) similar to the current study for patients discharged with TB from a tertiary-care hospital, but they did not report the causes of death. In a population-based nationwide cohort study, Christensen et al. (27) reported that patients with pulmonary TB had an increased risk of death from natural causes (infectious, malignant, respiratory, rheumatic, liver/pancreatic diseases, and diabetes) and from unnatural causes (alcohol and drug abuse, injury, and poisoning). The extrapulmonary TB patients had an increased risk of death due to infectious, gastrointestinal, and genitourinary diseases, diabetes, and gastrointestinal and hematologic neoplasms.

Cohort studies (28-31) have revealed that patients treated for TB have a substantially increased risk of death compared to the general population, even following apparently successful treatment. But neither of these studies has assessed post-discharge mortality. In a retrospective cohort study, Silva et al. (15) evaluated new cases of TB treated in the emergency room of a university hospital and that required hospitalization. They included 305 patients with TB and described an in-hospital mortality of 16.4% and one-year mortality of 31.8%. Therefore, the post-discharge mortality was 15.4%. They did not evaluate clinical factors associated with death. The current study included newly diagnosed TB (all forms) who were hospitalized for the disease and initiated TB treatment during hospitalization. Patients who died before being discharged from the hospital were excluded from the study. It should be noted that the median length of hospital stay was 19 days and, consequently, all the patients were at the beginning of treatment when discharged.

In this study, we demonstrated that older people with TB had higher mortality after hospital discharge. As previously described (32-34), elderly individuals with TB are more likely to have a non-specific clinical presentation of TB and are more likely to experience a delay in TB diagnosis and treatment, resulting in higher mortality. Older people had higher risk of TB-specific and non-TB-specific mortality. Also, higher mortality among older TB patients may be due to waning immunity and increased comorbidities.

The Charlson Comorbidity Index is a method of categorizing comorbidities of patients. Each comorbidity category has an associated weight (from 1 to 6). A score of zero indicates no comorbidities. The higher the score, the more likely the predicted outcome will result in mortality or higher resource use (35). In the present study, the Charlson Comorbidity Index was associated with TB mortality. Medical comorbidities interact with TB at multiple levels. The interaction of comorbidities and TB is complex. Several comorbidities that are prevalent in ageing populations may further increase the risk of developing active TB disease. (32) They may aggravate the TB process from latent to active or even to disseminated forms, cause diagnostic challenges, and lead to ineffective treatment. On the other hand, comorbidities may restrict the use of some potent anti-TB drugs. Furthermore, TB itself can aggravate or impair the diagnosis and management of comorbidities (35).

Fox et al. (31) reported that a high proportion of deaths after TB treatment was due to non-communicable diseases, such as stroke and cancer. Given the high proportion of patients who smoked and the older age of the cohort, enrollment in TB treatment provides an ideal opportunity to offer primary prevention for cardiovascular disease.

In the current study, the proportion of subjects with sputum was higher in those who died than in those who survived. Silva et al. (14) determined the prevalence of pulmonary TB using a symptom-based active case finding strategy in an emergency room of a public hospital. They reported that sputum production was found to be a negative predictor of pulmonary TB. The plausible explanation for that finding was that patients who could expectorate were diagnosed at a primary health care level, and those without sputum production were more difficult to diagnose. In our study, a possible explanation is that sputum production reflects greater TB lung injury or the presence of other chronic pulmonary disease.

The present study has some potential limitations. First, this was a single center study with a small sample size. Second, our institution provides free care for patients covered by the public health system. Consequently, the patient sample was biased toward the socially disadvantaged. Third, the study population was selected from patients referred to a reference center and was probably biased toward more severe disease. Fourth, GeneXpert MTB/RIF was not used for diagnosis, because it was not available during the study. This could lead to delay in diagnosis and in identification of rifampicin resistance, with a negative impact on prognosis.

Despite these concerns, this is one of the few studies that evaluated factors associated with mortality after hospital discharge, since most studies only assess in-hospital mortality. Older subjects, those who report sputum production, and those with a high comorbidity index should be followed-up more intensively after hospital discharge, with more regular appointments, since they may have a higher mortality during this period.

In conclusion, this prospective study evaluated post-discharge mortality in a cohort of adult subjects hospitalized for TB and who began treatment for the disease during hospitalization. Among them, 22.5% died within a median time of 3 months after hospital discharge. The mortality was higher in older subjects, in those who reported sputum production, and in those with a high comorbidity index. Most deaths were related to TB.
